# No dislocation rate gap between single and two-stage revisions with a cementless Dual Mobility Cup

**DOI:** 10.1051/sicotj/2025033

**Published:** 2026-03-03

**Authors:** Justine Bidard, Nory Elhadjene, Nicolas Zadel, Thomas Neri, Frédéric Farizon, Bertrand Boyer

**Affiliations:** 1 Orthopaedic and Traumatology Surgery, Saint-Etienne University Hospital, Jean Monnet University 42055 Saint-Etienne Cedex 2 France; 2 Department of Anaesthesia and Intensive Care, Saint-Etienne University Hospital, Jean Monnet University 42055 Saint-Etienne Cedex 2 France; 3 Interuniversity Laboratory of Motor Biology, EA 7424 42023 Saint-Etienne France; 4 Mines Saint-Etienne, INSERM, SAINBIOSE U1059 42023 Saint-Etienne France; 5 University of Lyon, Claude Bernard Lyon 1 University, Gustave Eiffel University, IFSTTAR, LBMC UMR_T9406 69675 Bron Cedex France; 6 Hospices Civils de Lyon, Orthopaedic and Traumatology Surgery Department, Edouard Herriot Hospital 69003 Lyon France

**Keywords:** Dual-mobility cup, Total hip arthroplasty, Revision, Dislocation, Two-stage

## Abstract

*Introduction*: A major complication of hip arthroplasty is dislocation. In revision, the rate of dislocation is even higher, especially among patients with hip prosthetic joint infection treated with two-stage surgery. The utility of a dual-mobility cup (DMC) in revision was already demonstrated but with a relatively low level of confidence due to the lack of direct comparison with other surgical techniques. We hypothesized that the dislocation rate for patients undergoing cementless DMC total hip arthroplasty (THA) would be similar between single and two-stage revisions. *Methods*: We conducted a single-center, retrospective, and case-control study from January 2011 through December 2020. During this period, 220 patients underwent a revision of their total hip arthroplasty. Among these, 40 patients experienced THA two-stage revision. This group constituted the cases in this case-control study. Each of the 40 cases was matched with 2 controls, single-stage surgery, on age, sex, and Paprosky grade, and we defined the groups according to primary endpoint: dislocation rate. *Results*: There was no significant difference in dislocation rate between two-stage and single-stage revisions (7.5% vs 3.8%, *p* = 0.40). In univariate analysis, auto-inflammatory disease and immunosuppressive agent use were risk factors for dislocation. There was no significant difference in dislocation-free survival (log-rank test, *p* = 0.40) or re-revision (log-rank test, *p* = 0.92) between single-stage and two-stage revision THA. At the end of follow-up, the mortality rate did not differ between the two groups. No chronic instability was noted at the last follow-up (80.4 ± 38.5 months) in both groups. *Conclusion*: The dislocation rate was similar between single and two-stage revision THA using DMC. Further studies are warranted to highlight the potential benefits of DMC in preventing dislocation in two-stage revision THA.

## Introduction

One of the main complications of Total Hip Arthroplasty (THA) remains dislocation [1]. In 1974, Professor Gilles BOUSQUET and engineer André RAMBERT invented the concept of dual mobility total hip arthroplasty. This innovation helped to reduce the dislocation rate of THA while increasing joint mobility in large retrospective cohorts, with a very low or even zero dislocation rate [2]. Currently, the use of a Dual Mobility Cup (DMC) is becoming more common, especially in patients with risk factors for instability [3].

Worldwide revisions of total hip arthroplasty (THA) are estimated at approximately 20,000 per year [4]. For revision procedures, the rate of dislocation is even higher and is estimated at 5% to 20% for all causes of revision [5]. Prosthetic Joint Infection (PJI) is another complication of hip arthroplasty, with an estimated rate of 0.25% to 2% [6]. In patients with hip PJI, the dislocation rate is even higher, approximately 10% [7], due to the need for debridement, which can increase the bone and soft tissue damage. It is generally accepted that two-stage revisions are more prone to dislocation than single-stage revisions [8]. Several other factors may contribute to an increased dislocation rate in revision THA, including abductor muscle deficiency, difficulty in correctly positioning the implant in a previously remodeled hip, the number of previous revisions, a small femoral head diameter, and a history of instability [5]. The role of a DMC in revision has been demonstrated, but with a relatively low level of confidence, in cemented DMC in cages [9] or in uncemented DMC in the case of chronically infected THA treated with two stages [10]. Various degrees of bone and soft tissue damage were important confounding factors as the series were either heterogeneous in nature when used in cages and/or did not have a control group of single-stage DMC when used in two-stage PJI.

To date, no study has compared dislocation rates between two-stage and single-stage DMC revisions, to evaluate if DMC could counterbalance the effect of the two-stage on instability.

We performed a case-control study in patients undergoing revision of THA with a DMC, two-stage (infected) versus single-stage (considered non-infected). Our objective was to evaluate the dislocation rate of the two-stage revision with DMC. We hypothesized that cementless DMCs would provide comparable dislocation rates between single- and two-stage revision THAs.

## Material and methods

### Study design

This study was a single-center, retrospective, and case-control study conducted between January 2011 and December 2020. The study protocol was approved by the local ethics committee (IRBN1422023/CHUSTE). Formal informed consent is not required for this type of study.

### Patients

**Figure 1:**
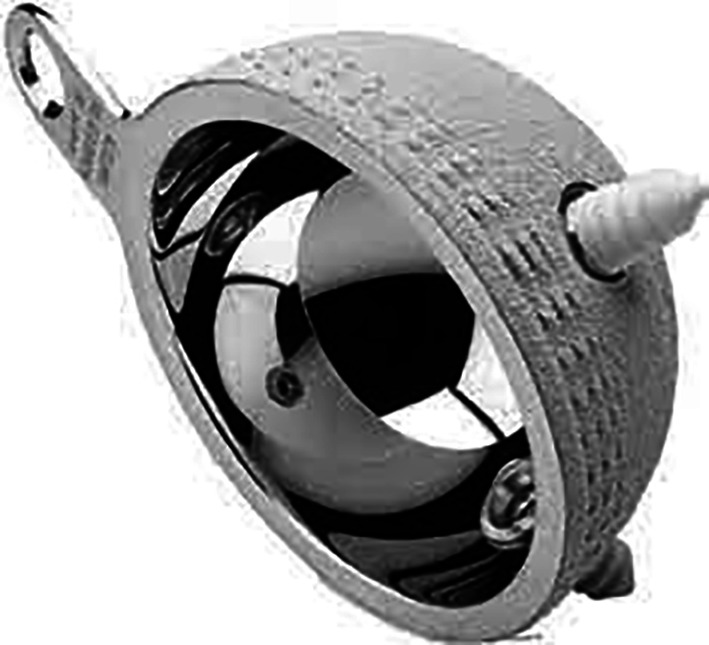
Dual-mobility cup Novae-E TH.

**Figure 2:**
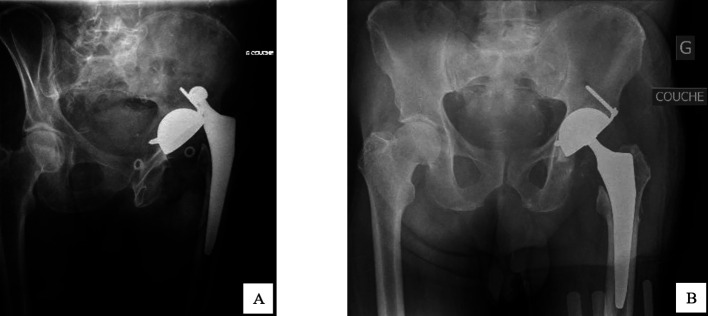
Radiographs (A) and (B) show dislocation and postoperative revision with DMC Novae-E TH, respectively.

The acetabular defect could introduce heterogeneity and could influence the dislocation rate [9]. Only patients with Paprosky 1 or 2A were considered because higher Paprosky grades often require additional reconstruction techniques such as bone grafting or reinforcement devices, which can alter implant stability and increase the risk of dislocation. In the period study, 489 THA with DMC Novae-E TH were performed (Figures 1 and 2); this DMC was suited for Paprosky 1 or 2A revisions; 220 patients of them were revision arthroplasties. Among these, 40 patients experienced a two-stage revision THA.

**Figure 3:**
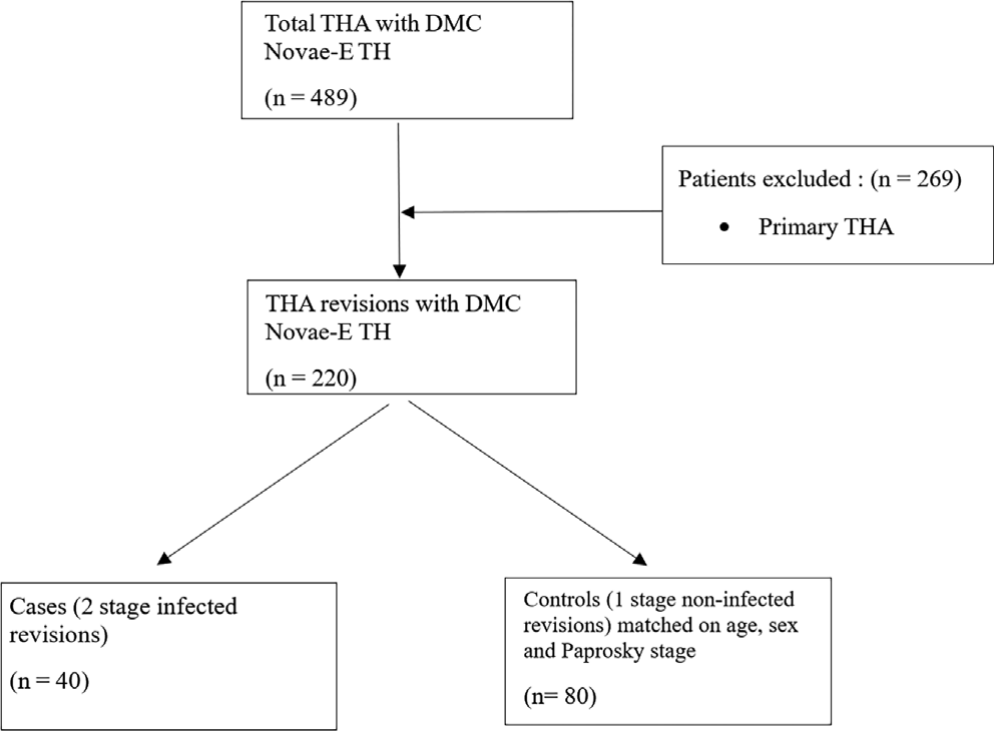
Flow chart study.

They constituted the group of cases in this case-control study. Controls were defined as single-stage revisions, matched on age, sex, and Paprosky grade. Each of the 40 cases was matched with two controls, and we defined the two groups according to the primary endpoint: dislocation rate. All data were resumed in the study flow chart (Figure 3). Comparing two-stage surgery for chronic infection to one-stage surgery for non-infected cases was considered a worst-case scenario in terms of instability, as infection is a known risk factor for instability. Therefore, this difference between cases and controls was not considered deleterious to the analysis.

Perioperative data were collected from the medical database. Perioperative data included: age, anthropometric data (height and weight to calculate body mass index) before surgery, American Society of Anesthesiologists (ASA) score, frailty using the Clinical Frailty Scale (CFS), comorbidities as neoplasia, diabetes mellitus and autoinflammatory disease, smoking and alcohol history, drug’s patients as antiplatelet therapy, anticoagulant or immunosuppressive agent, preoperative frailty using the CFS, residential facilities before surgery and death at last follow up. Minimum potential follow-up was 5 years after hip reimplantation.

### Clinical and radiological assessments

Clinical and radiological assessments were performed postoperatively at 45 days, 3 months, 6 months, 1 year, and every two years until the final follow-up. Re-revision was defined as any subsequent surgery required to treat a new dislocation after the initial revision. The radiological assessment focused on dislocation, fracture of prosthesis implant, aseptic cup or femoral stem loosening, leg length inequality, and prosthesis wear (Table 1). Instability was defined as the presence of recurrent dislocations confirmed by radiographic findings.

**Table 1:** Dual-mobility cup (Novae™) data.

Variables	Case group (*n* = 40)	Control group (*n* = 80)	*p*-value
Side			0.51
Right, *n* (%)	15 (37.5)	35 (43.8)	
Left, *n* (%)	25 (62.5)	45 (56.3)	
Femoral head diameter (mm), *n* (%)			0.05
22.2	40 (100)	72 (90.0)	
28	0 (0)	8 (10.0)	
Femoral offset (mm)	44.2 ± 10.5	44.3 ± 11.3	0.66
Cemented stem, *n* (%)	3 (7.5)	3 (7.5)	0.40
Acetabular bone defect areas, *n* (%)			0.46
0	37 (92.5)	68 (85.0)	
1	3 (7.5)	12 (15.0)	
Femoral bony defect areas, *n* (%)			0.42
0	29 (72.5)	48 (60.0)	
1	6 (15.0)	15 (18.8)	
2	2 (5.0)	13 (16.3)	
3	2 (5.0)	3 (3.8)	
4	1 (2.5)	1 (1.3)	

### Surgical data

General anesthesia was chosen for all patients. All revisions were performed via a posterolateral approach. Prosthetic Joint Infection diagnosis was set according to the Musculoskeletal Infection Society [11]. In the case group (septic revision group), the first procedure consisted in debridement, implant removal, thorough synovectomy, followed by pulsed lavage of at least 3 L of dilute Betadine^®^ then 3 L of saline solution and implantation of an antibiotic-loaded spacer (first OneStage™, Zimmer-Biomet, Warsaw, USA between 2011 and 2015, then Subiton™, Prothys Orthopédie, Brive-la-Gaillarde, France, between 2015 and 2020). Antibiotic therapy was initiated for a minimum of 6 weeks. Spacer dislocation was systematically documented but did not influence the planning of the second-stage procedure, even in cases where dislocation had occurred. The hip reimplantation, also defined as the second stage, was validated by a collegial committee (At least one physician from orthopedics, infectious diseases, and microbiology departments, from the Bone and Joint Infection Referral Centre) based on clinical and biological evidence of sepsis control, after antibiotics had been discontinued for at least 2 weeks (antibiotic holiday). At the final review, microbiological specimens were obtained, and the joint was again thoroughly irrigated. Antibiotic therapy was reintroduced after the sampling and subsequently re-evaluated according to the results of the microbiological sampling.

### Statistical analysis

Normal distribution was assessed using the Shapiro-Wilk test. Normally distributed quantitative variables were presented as means ± standard deviation. Quantitative variables not normally distributed were presented as medians and interquartile ranges. Student’s *t*-test or, if necessary, a Mann-Whitney test, was used to compare quantitative variables. Qualitative variables were presented as frequencies and percentages. A chi-square test or Fisher’s exact test, if necessary, was used to compare qualitative variables. Kaplan-Meier survivorship analyses were performed for several endpoints as re-revision, dislocation, and global survival of DMC. Confidence intervals of 95% (95% CI) were determined by Greenwood’s algorithm. The threshold for statistical significance was set at *p* < 0.05. Statistical analyses were performed with R (version 4.0.0) and R studio (version 4.2.1) software.

## Results

### Patients data

Overall demographic and clinical characteristics are summarised in Table 2. A total of 120 THA revisions were included in our study. The median duration of follow-up was 73 months (IQR 53–122) for the case group and 94 months (IQR 75–106) for the control group (*p* = 0.56). Death occurred at an average time of 39 months (SD 44 months) post-first surgery. The case group had a greater history of diabetes mellitus and inflammatory disease than the control group (*p* = 0.03 and *p* < 0.001, respectively). In the case group, all patients underwent revision for PJI. In the control group, the revision was required for several reasons: dislocation (6.5%), acetabular loosening (27.5%), femoral loosening (3.8%), implant failure (including periprosthetic fracture) (38.8%), polyethylene wear (15%) and other reasons (7.5%). All DMC data was resumed in Table 1. The distribution of femoral head diameters was found to differ between the two groups. A larger proportion of 28 mm diameters were found in the control group. However, head diameter does not influence jumping distance in the DMC.

**Table 2:** Baseline patient characteristics.

Variables	Case group (*n* = 40)	Control group (*n* = 80)	*p*-value
Age (years)	70.5 ± 18.3	74.0 ± 18.1	0.12
Gender (men), *n* (%)	22 (55.0)	44 (55.0)	1
Body mass index (kg m^−2^)	27.9 ± 6.1	26.5 ± 5.7	0.14
ASA score (1/2/3/4), *n*	5/18/15/2	10/39/30/1	0.66
Clinical Frailty Scale score	2 [1–5]	3 [1–5]	0.425
Smoking history, *n* (%)	7 (17.5)	9 (11.3)	0.34
Alcohol history *n* (%)	6 (15.0)	8 (10.0)	0.42
Neoplasia history *n* (%)	1 (2.5)	12 (15.0)	0.06
Diabetes mellitus history *n* (%)	13 (32.5)	12 (15.0)	0.03
Autoinflammation disease *n* (%)	11 (27.5)	3 (3.8)	< 0.001
Anticoagulant use, *n* (%)	8 (20.0)	15 (18.8)	0.87
Anti-Platelet therapy use, *n* (%)	8 (20.0)	25 (31.3)	0.19
Immunosuppressive agent use, *n* (%)	3 (7.5)	1 (1.3)	0.11
Residential facilities, *n* (%)	3 (7.5)	8 (10.0)	0.75
Follow-up (months)	73 ± 69	94 ± 31	0.56

### Dislocation and postoperative data

There was no significant difference in dislocation rate between two and single-stage revisions (7.5% vs 3.8%, *p* = 0.40) (Table 3). In the two-stage group, one patient needed open surgical revision. All other dislocations in the two groups were treated without open surgery, under general anesthesia. Only one patient had a recurrent dislocation 3 months after the first dislocation and did not present an instability afterwards. There was no femoral loosening or prosthesis implant failure in the postoperative period up to the end of the follow-up. Only one cup of aseptic loosening was recorded. No prosthesis wear was observed. Mortality rate at the end of follow-up did not differ between the two groups (20% vs 28.8%, *p* = 0.30).

**Table 3:** Postoperative complications after revision.

Variables	Case group (*n* = 40)	Control group (*n* = 80)	*p*-value
Dislocation, *n* (%)	3 (7.5)	3 (3.8)	0.40
Fracture of prosthesis implant, *n* (%)	0 (0)	0 (0)	1
Aseptic cup loosening, *n* (%)	1 (2.5)	0 (0)	1
Femoral stem loosening, *n* (%)	0 (0)	0 (0)	1
Leg length inequality (mm)	2.2 ± 4.4	0.1 ± 2.7	0.10
Radiologic prosthesis wear, *n* (%)	0 (0)	2 (2.5)	0.55
Mortality, *n* (%)	8 (20.0)	23 (28.8)	0.30

### Implant survival

**Figure 4:**
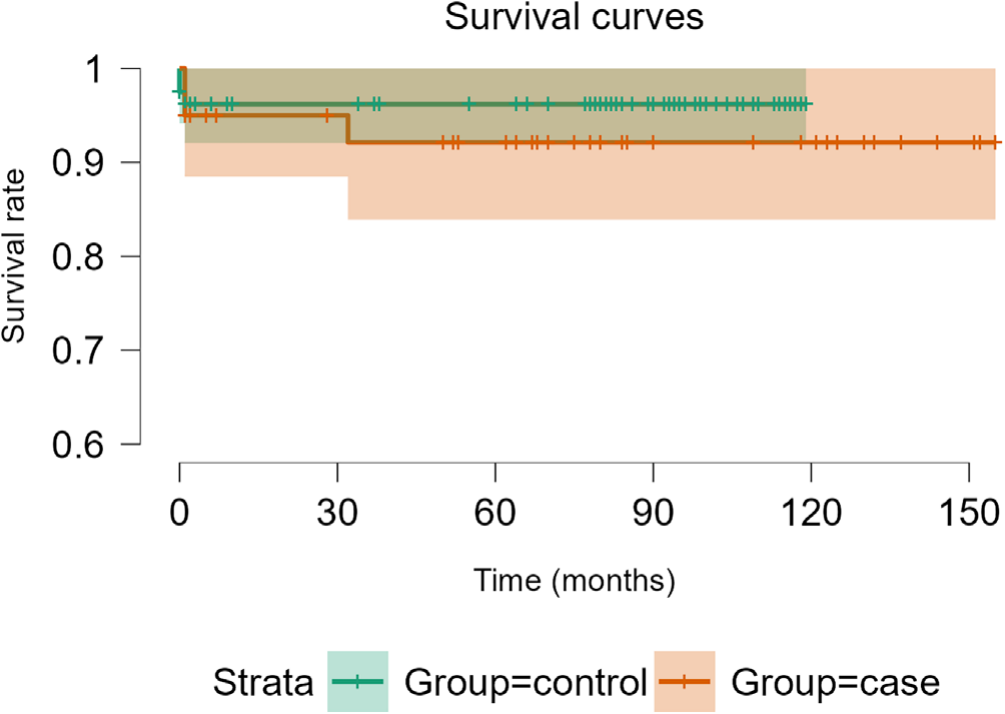
Survivorship Free of dislocation after THA revision with DMC Novae-E TH.

**Figure 5:**
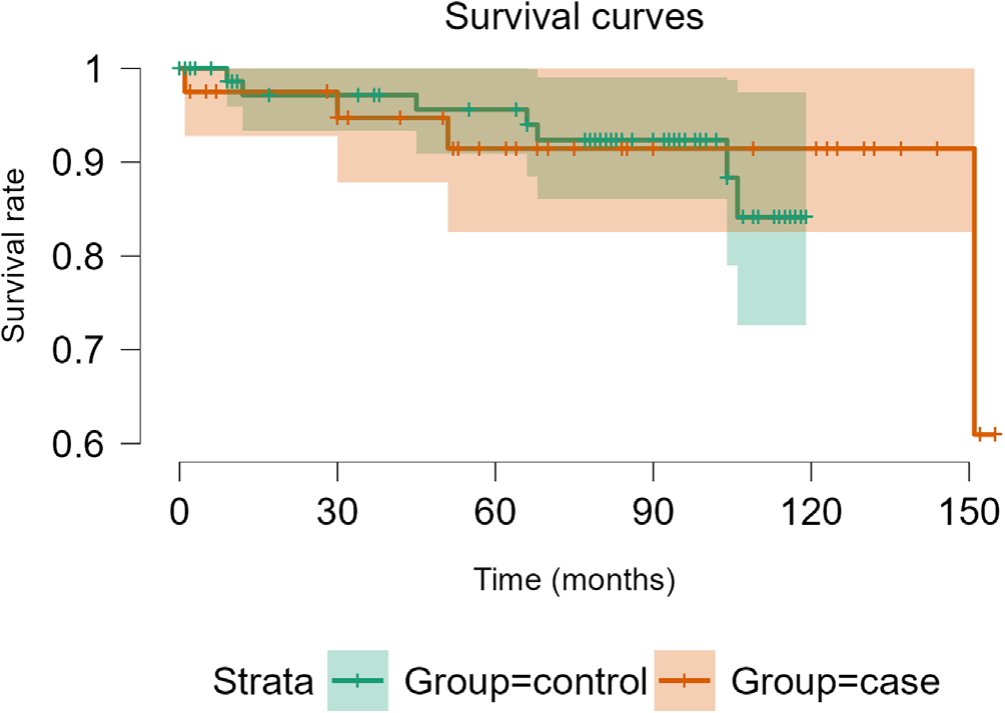
Survivorship Free of re-revision after THA revision with DMC Novae-E TH.

In the case group, a total of 36 patients were not revised, and 3 patients had a dislocation at the last follow-up; 7 patients died without a dislocation (17.5%), and 6 patients died without being revised (15.0%). Of the 4 re-revision patients (10.0%), one had a new case of PJI, one had an intra-prosthetic dislocation [12] requiring open surgical re-revision, one hip was revised for cup aseptic loosening, and one had an early revision with implant retention for compressive hematoma. The survival rate with dislocation as the endpoint was 95.0% [95% CI 88.5–100] at 20 months and 92.1% [95% CI 83.9–100] at 140 months of follow-up (Figure 4, orange line data). The survival rate considering re-revision as the endpoint was 94.7% [95% CI 87.8–100] at 40 months and 91.4% [95% CI 82.6–100] at 140 months of follow-up (Figure 5, orange line data).

In the control group, a total of 77 hips did not dislocate, and 7 patients experienced a new revision of their hip at the last follow-up; among these 7 re-revisions, 3 patients had a peri-prosthetic fracture, 3 patients had a new PJI, and one patient had a drainage of compressive hematoma. The survival rate considering dislocation as the endpoint was 96.2% [95% CI 92.1–100] at 60 and 100 months of follow-up (Figure 4, green line data). The survival rate with re-revision as the endpoint was 95.6% [95% CI 90.9–100] at 60 months and 92.3% [95% CI 86.1–99.0] at 100 months of follow-up (Figure 5, green line data).

No patient had radiographic evidence of femoral or acetabular loosening at the final follow-up.

There was no significant difference in dislocation-free survival (log-rank test, *p* = 0.40) or re-revision (log-rank test, *p* = 0.92) between single-stage and two-stage revision THA.

### Influence of different factors on dislocation

In our global cohort, we counted only 6 dislocations (3 dislocations in each group). In univariate analysis, autoinflammatory disease and immunosuppressive agent use were risk factors for dislocation (*p* = 0.02 and *p* < 0.001, respectively). The spacer dislocation was a risk factor for dislocation (*p* = 0.01). There was a statistical trend for frailty (defined by CFS or residential facilities) as a risk factor for dislocation (Table 4).

**Table 4:** Risk factors of dislocation.

Variables	Dislocation (*n* = 6)	No dislocation (*n* = 114)	*p*-value
Age (years)	80 ± 5.8	74 ± 13.8	0.21
Gender (men), *n* (%)	4 (66.7)	50 (43.9)	0.41
Body mass index (kg m^−2^)	28.0 ± 4.3	26.0 ± 5.9	0.98
ASA score (1/2/3/4), *n*	1/1/4/0	14/56/41/3	0.40
Clinical Frailty Scale score	3 [2–5]	3 [1–5]	0.07
Smoking history, *n* (%)	0 (0)	16 (14.0)	1
Alcohol history, *n* (%)	0 (0)	14 (12.3)	1
Neoplasia history, *n* (%)	1 (16.7)	12 (10.5)	0.51
Diabetes mellitus history, *n* (%)	2 (33.3)	23 (20.2)	0.60
Autoinflammation disease, *n* (%)	3 (50.0)	11 (9.6)	0.02
Anticoagulant use, *n* (%)	2 (33.3)	21 (18.4)	0.32
Anti-Platelet therapy use, *n* (%)	2 (33.3)	31 (27.2)	0.66
Immunosuppressive agent use, *n* (%)	3 (50.0)	1 (0.9)	< 0.001
Residential facilities, *n* (%)	2 (33.3)	9 (7.9)	0.09
Spacer dislocation	2 (33.3)	2 (1.8)	0.01

## Discussion

The dislocation rate after THA revision remains a challenging surgical problem, especially in patients with a history of hip PJI [13]. In our study, it was hypothesized that the dislocation rate in patients undergoing total hip arthroplasty with cementless DMC would be similar for one-stage and two-stage revisions. Our hypothesis was confirmed; our cases had a similar dislocation rate to that of the controls. Only one patient had an early recurrent dislocation, treated with implant revision; no patient was revised for instability. Autoinflammatory disease and immunosuppressive agent use were the only risk factors found, ASA score was not found to be a risk factor, and there existed only a statistical trend for frailty.

Regarding two-stage surgeries without DMC, Hartman and Garvin, and Berend et al. showed a high dislocation rate of 15 and 21.4%, respectively, in their studies [13, 14]. The cup types in their cohorts were all “single mobility” standard cups or S-ROM™ [13, 14]. The dislocation rate of our cohort was similar to those found in previous studies using DMC with a posterolateral approach in single-stage revision [15].

Furthermore, Unter Ecker et al. showed a higher rate of dislocation and revision in patients who underwent single-stage, complex revision THA on infected patients with DMC [16]. This higher dislocation rate may be attributed to the indications for the surgical index procedures in which dual mobility cups were used, closer to the high Paprosky stages and the corresponding higher dislocation rate of the Sayac et al. study [9], and also to the aggressive debridement technique advocated by the EndoKlinik, allowing them to offer single-stage surgery on most patients but possibly at the expense of increasing the dislocation rate due to soft tissue damage, especially on abductor muscles, that could decrease joint stability.

In addition, risk factors for dislocation after THA revisions were not comparable to those after primary THA [17]. Many risk factors in PJI have been studied previously. In our study, spacer dislocation was a risk factor for dislocation. Garceau et al. and Chalmer et al. showed a statistical association between spacer dislocations and dislocations after two-stage infected revision [18, 19]. This can be explained by the fact that the risk factors for dislocation are the same for spacers or THA. On the other hand, spacer instability may lead to inadequate lateral superior acetabular coverage [20].

In our work, other risk factors were found: chronic inflammatory arthropathy and immunosuppressive therapy. However, many patients (28.6%) with chronic inflammatory arthropathy were treated with immunosuppressive therapy, so this is one of the confounding factors. However, chronic inflammatory arthropathy has been implicated as a risk factor for primary THA [21].

Our study has some limitations. First, it was an observational, non-randomized, and retrospective case-control study. To reduce this bias, two groups were matched on age and sex.

The absence of a statistical difference in dislocation rate may be linked with an underpower of our study, but even if there was a difference that could be proven by a larger study, cementless DMC use at least decreased this difference between singel-stage and two-stage revisions, not visible in a fair number of patients. Finally, another limitation of our study is the short follow-up. Previous very long follow-up studies on DMC have not shown an increase in dislocation rate with follow-up time. The relatively short follow-up may have underestimated late dislocations, although prior studies have not shown increasing risk over time [2, 22]. A 10-year follow-up update is already planned for this cohort on this secondary objective.

The dislocation rate gap was similar between single and two-stage revision THA using DMC. Larger studies are warranted to highlight the potential benefits of DMC in preventing dislocation in two-stage revision THA. In the future, the use of DMC could be considered when a two-stage procedure is required.

## Data Availability

Blinded data from this study are available on request.
